# Distributed Channel Allocation and Time Slot Optimization for Green Internet of Things

**DOI:** 10.3390/s17112479

**Published:** 2017-10-28

**Authors:** Kaiqi Ding, Haitao Zhao, Xiping Hu, Jibo Wei

**Affiliations:** 1 College of Electronic Science, National University of Defense Technology, Changsha 410073, China; dingkaiqi15@nudt.edu.cn (K.D.); wjbhw@nudt.edu.cn (J.W.); 2 Shenzhen Institutes of Advanced Technology, Chinese Academy of Sciences, Shenzhen 518055, China; 3 Department of Mechanical and Automation Engineering, Chinese University of Hong Kong, Hong Kong

**Keywords:** energy saving, channel allocation, time slot optimization

## Abstract

In sustainable smart cities, power saving is a severe challenge in the energy-constrained Internet of Things (IoT). Efficient utilization of limited multiple non-overlap channels and time resources is a promising solution to reduce the network interference and save energy consumption. In this paper, we propose a joint channel allocation and time slot optimization solution for IoT. First, we propose a channel ranking algorithm which enables each node to rank its available channels based on the channel properties. Then, we propose a distributed channel allocation algorithm so that each node can choose a proper channel based on the channel ranking and its own residual energy. Finally, the sleeping duration and spectrum sensing duration are jointly optimized to maximize the normalized throughput and satisfy energy consumption constraints simultaneously. Different from the former approaches, our proposed solution requires no central coordination or any global information that each node can operate based on its own local information in a total distributed manner. Also, theoretical analysis and extensive simulations have validated that when applying our solution in the network of IoT: (i) each node can be allocated to a proper channel based on the residual energy to balance the lifetime; (ii) the network can rapidly converge to a collision-free transmission through each node’s learning ability in the process of the distributed channel allocation; and (iii) the network throughput is further improved via the dynamic time slot optimization.

## 1. Introduction

The ubiquitous presence of devices combined with intelligent sensing [1,2], low-power operation [3,4], and communication ability [5,6] enable the rapid expansion of the Internet of Things (IoT). Additionally, the IoT delivers a reliable, efficient, and greener energy transmission to better support services in sustainable smart cities. The challenges of IoT involve energy consumption, bandwidth requirement dynamic communication environment, and etc. Regarding energy consumption, the most severe challenge is that nodes are significantly influenced by limited battery capacity and it is difficult to replace or recharge their batteries in run-time. Thus, effective power management is very important in IoT [7], e.g., so that it can monitor the components of energy consumption and dynamically switch the state of each node accordingly.

Currently, most algorithms of power management focus on constrained optimization problem to minimize power consumption under performance constraints [8,9]. However, in the distributed network, each node is difficult to autonomously save energy without any guideline. Moreover, the power consumption in anti-interference and data retransmission will increase sharply with the expansion of the devices. Multi-channel communication, which allows simultaneously transmissions along multiple non-overlap channels, is a promising approach to reduce interference. Nevertheless, there are two research challenges in multi-channel communications that have not been addressed by the existing research need to be explored: (i) Most channel allocation processes ignore the problem of energy consumption varies on different channels; and (ii) Less attention is laid in the channel quality.

To address these two challenges, we design a solution that jointly considers channel allocation and time slot optimization. In our solution, each node will learn and make decision based on its own local information in a distributed manner, without requiring information exchanges and time synchronizations between neighboring nodes. The energy saving is a joint optimization in both frequency domain and time domain, i.e., via channel allocation and time slot adaptation. The benefits for this are two-fold. First, in frequency domain, we employ channel allocation to save energy. As the channel allocation plays an important role in two aspects: (i) it can enable each node simultaneously transmits along different channels to alleviate interference and reduce wasteful energy in anti-interference and data retransmission; (ii) it can allocate an appropriate channel with lower energy consumption to node. Second, in time domain, we employ the time slot optimization to save energy. In our proposed scheme, the sleeping time and spectrum sensing time are adaptive and always keep the optimal values. This optimized adaptation has two significant advantages: (i) the optimal sensing time yields the highest throughput. On one hand, too short sensing time cannot successfully detect the channel state, and thus transmission collisions diminish the throughput. On the other hand, too long sensing time will consume more energy and thus left less energy for data transmission, which also diminish the throughput; (ii) the optimal sleeping time prolongs the lifetime of nodes. If the sleeping time is too short when deals with the increasing traffic arrival rate, it will drain the energy much faster and thus cannot guarantee the lifetime of nodes. On the contrary, if the sleeping time is too long, it cannot guarantee the throughput. Therefore, the optimal solution can not only control the power consumption but also maximum the normalized throughput.

The paper is organized as follows: Section 2 gives an overview of related work about energy management in IoT. Section 3 summarizes the distributed channel allocation and time slot optimization algorithms, and presents the transmission structure of each node. Section 4 proposes a channel ranking algorithm so that each node can rank its available channels based on the channel condition. Section 5 proposes a distributed channel allocation algorithm that each node can choose proper channel based on the channel ranking and its own experience. Section 6 analyzes the data transmission rate and derives the optimal solution. Section 7 presents the results of our experiments for each of the aforementioned cases. Section 8 concludes this paper.

## 2. Related Work

Power efficiency plays an extremely important role in the rapid development of IoT, and the challenge of the power efficiency is how to guarantee the network performance while minimizing the energy consumption. In the literature, various power management algorithms have been proposed, which can be broadly classified into two categories: power control and time slot control. 

Dynamic power management (DPM) [10] is proposed to selectively power off the idle part to save energy, but the disadvantage of this approach is that it requires complete knowledge of past and future workloads to fulfill the target. The power control algorithm (PCA) in [11] controls the transmission power consumption according to different channel conditions. PCA can improve the overall energy efficiency, but the coordination between nodes is done implicitly, and thus a part of energy is consumed by beacon exchange. As for time slot control, energy consumption in sleeping period is much lower than data transmission period [12], and this implies it is worthwhile for nodes to make certain trade-off to balance the time between sleeping period and transmission period. Studies [13,14] propose that nodes can choose to switch into sleeping mode when there is no data to be received or transmitted. In [15], the algorithm balances the energy consumption and end-to-end delay by appropriately scheduling the sleeping time. Another important time control is about spectrum sensing period. Insufficient spectrum sensing period will lead to imperfect channel state information, while excessive spectrum sensing period will decrease the time for data transmission [16]. Both the sleeping period and spectrum sensing period impact the network performance and thus need to be jointly optimized.

The aforementioned algorithms can be applied to save the energy consumption effectively, but it still cannot radically deal with energy consumption if the interference is caused by the expansion of the network. Therefore, multi-channel communication is introduced to reduce the network interference [17]. In multi-channel communication, the channel allocation plays a crucial role, which can be achieved via two methods: cooperative method and non-cooperative method. Cooperative method can learn from the exchange of information among nodes, while non-cooperative method can learn by nodes’ own past experience [18]. In [19], a channel assignment with power constraint is proposed to maximize the total throughput. However, this method needs to exchange control signals and each node cannot totally decide its behavior in a distributed manner. The information exchange has to occupy certain time slots and energy so that the node’s life is shortened. Therefore, nodes are better to decide their behaviors by themselves without too much information exchange for negotiation. Another important problem for channel allocation is that most researches are based on the assumption that all channels are identical. In practice, in multi-cell OFDM systems, the sub-channels will be reused by nodes, which may cause interference to each other, moreover, different sub-channels may suffer from different noises and have various bandwidth, resulting in different transmission energy consumption. Therefore, study [11] adopts a closed-loop power-control (CLPC) algorithm, which adjusts the transmission power according to different channel conditions. Moreover, authors in [20] propose a channel allocation scheme considering the signal to noise ratio (SNR) of each channel. And then more research is conducted considering the effect of the channel width [21,22]. Different channel quality leads to different transmission power [11]; therefore, it is critical to allocate the high-quality channel to the node with lower residual energy to balance the node lifetime.

Most existing contributions on energy saving consider either only the impact of power management or only the impact of channel allocation. This paper aims to improve energy efficiency from both channel selection and time slot optimization. We first propose a distributed channel ranking algorithm so that each node can be allocated with ideal channel (e.g., the channel of higher rank in the channel ranking), while considering different properties of channels, including bandwidth, signal-to-interference-plus-noise ratio (SINR), coherent bandwidth, coherent time and channel energy consumption. We also enable each node to learn from history knowledge for better performance on convergence and scalability. After the channel has been determined, we jointly optimize the sleeping period and spectrum sensing time to maximize the data transmission rate while guaranteeing that the lifetime of nodes can reach the expected survival time. Specifically, the main contributions of our paper are summarized as follows:

- We propose a novel solution that considers the complementary relations between channel allocation and time slot design for both energy saving and interference alleviating.
- We present a channel allocation algorithm that takes channel conditions into consideration and involves learning ability. It can not only allocate the most ideal channel to each node, but also enable the channel allocation achieves fast collision-free convergence.
- Through optimal time slot adaptation, our solution can maximize normalized throughput while satisfying power constraint and guaranteeing the lifetime of the nodes.
- Our solution is easy to implement. It requires no central coordination, global information, or information exchange/time synchronization among nodes. Each node can operate solely based on its own local information in a distributed manner.

## 3. System Model

**Figure 1:**
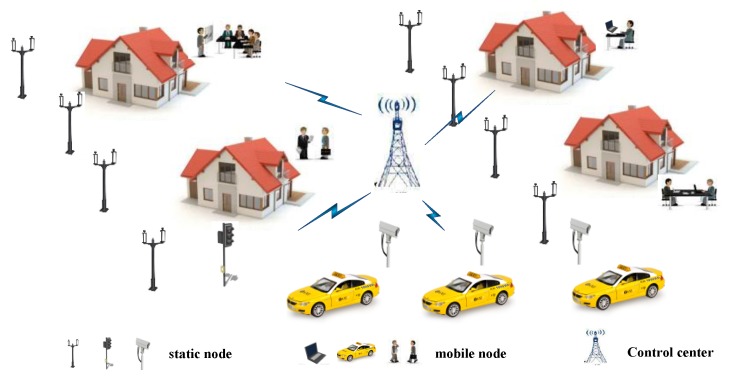
System model of high-density Internet of Things (IoT)*.*

This paper focuses on data-gathering/data-collection applications of IoT, where the many-to-one/converge-cast communication model is applicable as shown in Figure 1. In this network, spatially distributed nodes monitor various physical and environmental factors, and deliver the acquired information to the control center. The control center is responsible for managing the whole collection. The framework adopts Orthogonal Frequency Division Multiplexing (OFDM) in physical layer. The spectrum band of 5 GHz is slipped into m (m=16) channels. We consider the frequency-selective Rayleigh fading channel models. We assume the network design using short range radio standards that can be used to develop a wide area networks to support large number of IoT devices for various applications in a city environment [23]. The results of this paper are also transferable to lower frequencies, e.g., Low Power Wide Area Network (LPWAN) technologies as well. As the paper focuses on optimization design for medium access control (MAC) protocol in high-density IoT network, it aims to improve energy efficiency through both channel allocation and time slot optimization. 

We adopt the channel hopping technique to alleviate interference. The channel hopping technique has been embraced by a number of technologies and standards. One of the most notable examples, the IEEE 802.15.4e Task Group [24] adopts the time slotted channel hopping (TSCH) technique to enhance the existing IEEE 802.15.4 standard. In our solution, each node is free to switch among these *m* channels, but can only use one channel to transmit at a time. When two nodes are within the interference range, collision will occur if they transmit on the same channel simultaneously.

In our system model, we consider computation offloading as an effective approach to solve the energy saving problem. In traditional IoT network, each central station should be able to support a massive number of IoT devices, which bring high computation burden to the central station in dense networks. Therefore, we adopt computation offloading technique to enable distributed nodes afford parts of the computation from the control center to alleviate its computation burden [25]. We allow each node to make decision on (i) the transmit channel to obtain a collision-free channel allocation; (ii) the optimal time including sleeping time and spectrum sensing time.

**Figure 2:**
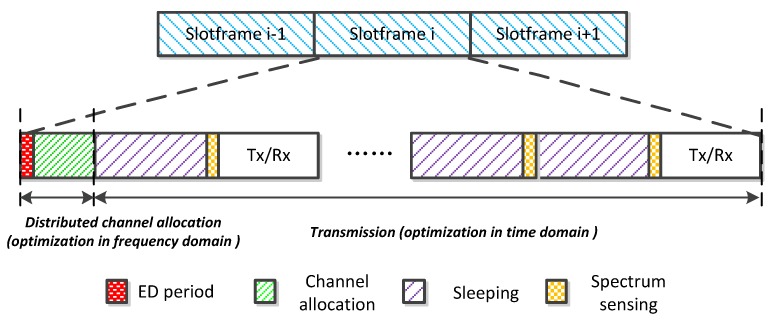
Transmission structure of the node*.*

The transmission structure of each node is described in Figure 2. We consider that time is divided into fixed length slotframes, and each slotframe is composed of 100 timeslots. Each slotframe has two processes: channel allocation and transmission. In the channel allocation process, the quality of channels need be firstly measured by Energy Detections (ED) [26], because the network conditions vary over time. ED can detect energy from variable-bandwidth channels. As the variable bandwidth will suffer different degree of noise power, thus a spectrum analyzer with different low pass filters is needed to measure the noise. Then, we update the available channel set (ACS) of each node based on the rank of channel condition. The node will dynamically hop between channels according to ACS until it switches on an empty channel, and then start to send HELLO packet periodically to dwell on this channel. In the transmission process, as the node has chosen the channel, it will adjust variable timeslots of sleeping and spectrum sensing based on the residual energy to ensure the transmission rate. If the selected channel is sensed idle, a transmission starts. Otherwise, the node has to continue to sleep. The node may go through successive sleeping and spectrum sensing before data transmission.

**Figure 3:**
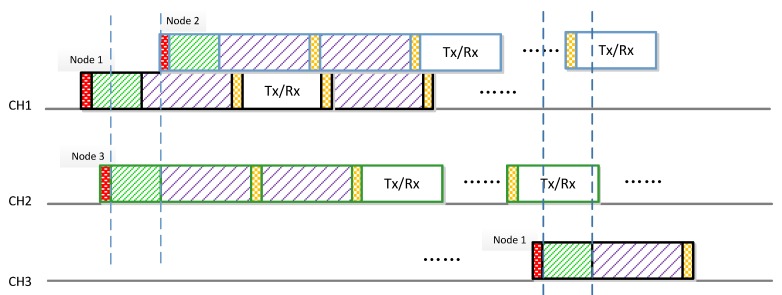
Desynchronization structure of the node*.*

Furthermore, as the network does not need synchronization, the following scenario may occur as shown in Figure 3. Although node 1 and node 3 start channel allocation at different time slot, they will try to dwell on different channels to avoid interference, because their channel allocation periods overlap. However, node 2 starts channel allocation while the others two nodes (node 1 and node 3) finish the channel allocation. Since node 1 sleeps on CH1, node 2 considers CH1 as idle and thus dwells on CH1. Therefore, in order to avoid transmission collision, both node 1 and node 2 need to sense spectrum before transmission. If the channel is sensed busy, the node will continue to sleep until the channel is idle. Note that during the transmission period, nodes will not hop between channels, they will dwell on the allocated channel. When the next slotframe comes, they will hop again to achieve a collision-free channel allocation. For example, node 1 switches to an idle channel (e.g., CH3) to avoid interference with node 2 when the next channel allocation period arrives.

## 4. Channel Ranking Algorithm

The channel ranking algorithm aims to select the most desirable channel from all the available channels. Based on existing research [11,27,28,29], the important factors that affect channel conditions include bandwidth, Signal to Interference plus Noise Ratio (SINR), coherent bandwidth, coherent time and energy consumption. As the transmitter is capable to know which channel to hop and how many subcarriers can employ, thus the bandwidth of each channel is known and we assume the bandwidth is variable in 200 kHz–1 MHz. The other factors can be obtained via sending probe packets as proposed in [29].

- Bandwidth is an important attribute that affect wireless transmission in terms of rate and range. Moreover, applications require different bandwidth. For example, status indicators for temperature will send a very small amount of data, while camera sensors that transmit a video stream will send a much greater amount of data [23].
- SINR is used to give theoretical upper bounds on the channel capacity.
- The coherence bandwidth is a statistical measurement of the range of frequencies. It can be reasonably assumed that the channel is flat if the coherence bandwidth is greater than the data signal bandwidth.
- The coherence time is the time duration over which the channel impulse response is considered to be constant. Such channel variation is important in wireless communications systems, considering the Doppler effect.
- The energy consumption on difficult channel mainly depends on the transmission power. Moreover, the transmission power varies according to different channel conditions based on the closed-loop power-control (CLPC) algorithm [11]. CLPC can dynamically degrade the transmission power in a flat channel to save energy and increase the transmission power to compensate for the fading channel.

Inspired by the Multiple Attribute Decision Making (MADM) algorithm [30], we propose a channel ranking algorithm that each node will rank its available channels based on the aforementioned five attributes. First of all, we need to identify the weight of each attribute through pairwise comparisons. And then we can rank channels via analyzing the closeness of each channel to the best channel condition.

### 4.1. Identify the Weight of Each Attribute

The weights of diversified attributes are determined by the following three steps.

(1) Construct the attribute comparison matrix

We first construct a matrix for the attribute comparison as follows,

(1) $$B={\left(b_{ij}\right)}_{n\times n}=\left[\begin{array}{ccc} b_{11} & \cdots & b_{1n}\\ \vdots & \ddots & \vdots \\ b_{n1} & \cdots & b_{nn} \end{array}\right]$$

where *n* is the number of attributes, and $b_{ij}$ represents the relative importance of Attribute i over Attribute j. Assuming Attribute i is no less important than Attribute j, the value of $b_{ij}$ is defined in Table 1 [30] (note that $b_{ji}=1/b_{ij}$).

(2) Determine the weight matrix

To simplify the computation, we further normalize the comparison matrix B into $R={\left(r_{ij}\right)}_{n\times n}$, where $r_{ij}=\frac{b_{ij}}{\sum_{i=1}^{n}b_{ij}}\;\forall i\in \left[1,n\right],\;j\in \left[1,n\right]$. 

And then the weight of Attribute *i* (denoted by $w_{i}$) can be calculated as the average of the *i*th row in the normalized comparison matrix R, namely $w_{i}=\frac{1}{n}\sum_{j=1}^{n}r_{ij}$. Therefore, the weight matrix W is a n×1 matrix, where $W={\left(w_{i}\right)}_{n\times 1}$. Note that $w_{i}$ represents the sum of the comparisons between attribute *i* with all attributes, and it is easy to prove that $\sum_{i=1}^{n}w_{i}=1$. 

(3) Conduct the consistency analysis

In the end, we do consistency analysis to validate the effectiveness of the weight. 

Our goal is to compute a vector of weights $W={\left(w_{i}\right)}_{n\times 1}$ associated with matrix B. However, the attribute comparison only represents the referee’s preferences with subjectivity, pairwise comparison matrix can be not absolutely consistent. Two cases will lead to it. In the first case, it is a contradictory matrix. In the second case, the matrix B is neither totally consistent nor contradictory. In this case, Saaty [27] defined the consistency index (CI) as follows. CI reflects the consistency of pairwise comparisons. We first construct consistency vector (CV) through multiplying the pair-wise comparison matrix B by the corresponding weight W as follows

(2) $$\mathrm{CV}=B\cdot W={\left(b_{ij}\right)}_{n\times n}\cdot {\left(w_{i}\right)}_{n\times 1}$$

Define $\lambda_{max}$ as the maximum feature value of comparison matrix B and it can be computed as

(3) $$\lambda_{max}=\frac{1}{n}\cdot \left(\overset{n}{\underset{i=1}{\sum }}\frac{\sum_{j=1}^{n}cv_{ij}}{w_{i}}\right)$$

Then we need to calculate the CI according to [27]:

(4) $$\mathrm{CI}\;=\frac{\lambda_{max}-n}{n-1}$$

Smaller CI represents that the comparison matrix will be more likely to be consistent. On the contrary, larger CI means that the comparison matrix will deviate from the consistency. And Saaty [27] suggests that if CI > 0.10 the comparison matrix is not consistent enough and need to go back and revise the pairwise comparisons.

### 4.2. Rank the Channel

With the five attributes of each channel and the weight of each attribute, we can further construct an evaluation matrix to rank the channel. The detailed process of the channel ranking also comprises three steps.

(1) Construct the evaluation matrix

The evaluation matrix $X={\left(x_{ij}\right)}_{m\times n}$ should cover m channels and their respective n attributes (n=5 in this research). In order to eliminate the incommensurability of the evaluation matrix X, each attribute $x_{ij}$ needs to be normalized into the corresponding comparable element. The normalized evaluation matrix is $Y={\left(y_{ij}\right)}_{m\times n}$, where $y_{ij}=\frac{x_{ij}}{\sqrt{\sum_{i=1}^{m}x_{ij}^{2}}}\;\forall i\in \left[1,m\right],\;j\in \left[1,n\right]$.

And then, the weighted evaluation matrix can be calculated as $V={\left(v_{ij}\right)}_{m\times n}=W\cdot Y$.

(2) Calculate the “distances” of channel conditions

Define $V^{+}$ and $V^{-}$ respectively as the best and worst channel conditions, and they can be calculated as follows

(5) $$V^{+}=\left\{\langle \min(v_{ij}|i\in [1,m])|j\in j^{-}\rangle ,\langle \max(v_{ij}|i\in [1,m])|j\in j^{+}\rangle \right\}=\left\{v_{j}^{+}|j\in [1,n]\right\}$$

(6) $$V^{-}=\left\{\langle \max(v_{ij}|i\in [1,m])|j\in j^{-}\rangle ,\langle \min(v_{ij}|i\in [1,m])|j\in j^{+}\rangle \right\}=\left\{v_{j}^{-}|j\in [1,n]\right\}$$

where $j^{+}$ associates with the positive attributes like bandwidth, SINR, coherent bandwidth and coherent time, which mean that the higher value of these attributes, the better the channel condition will be. For instance bandwidth, SINR, coherent bandwidth and coherent time are all positive attributes. $j^{-}$ associates with the negative attributes like the channel energy consumption. Let $D_{i}^{+}$ be the distance of the channel i to the best channel condition, and $D_{i}^{-}$ be the distance of Channel i to the worst channel condition, we have

(7) $$D_{i}^{+}=\sqrt{\sum_{j=1}^{n}{\left(v_{ij}-v_{j}^{+}\right)}^{2}}\;\forall i\in [1,m],j\in [1,n]$$

(8) $$D_{i}^{-}=\sqrt{\sum_{j=1}^{n}{\left(v_{ij}-v_{j}^{-}\right)}^{2}}\;\forall i\in [1,m],j\in [1,n]$$

(3) Channel ranking

We define the relative distance of Channel *i* as follows,

(9) $$rd_{i}=\frac{D_{i}^{-}}{D_{i}^{-}+D_{i}^{+}}\;\forall i\in [1,m]$$

This relative distance represents how far the channel condition is from the worst condition while also considering its closeness to the best condition. Note that $rd_{i}\in \left[0,1\right],\;\forall i\in \left[1,m\right]$, and $rd_{i}=1$ means Channel *i* has the best condition and $rd_{i}=0$ means Channel *i* has the worst condition. The key motivation of the paper is to allocate the channel of high rd to respective nodes. Therefore, we can rank the channel in descending order of $rd_{i}$, ∀i∈[1,m].

## 5. Distributed Channel Allocation Algorithm

### 5.1. Basic Idea

After we establish the available channel set (ACS) according to the channel ranking, any node will switch onto the channel sequentially in the ACS and listen for a period T. During this period, if the node receives no HELLO packets from other nodes, it will dwell on this channel. Then it starts to send HELLO packet periodically (i.e., every T seconds) to claim dwelling on this channel. Otherwise, if it receives any HELLO packet from other nodes, it will switch its radio to the next free channel in ACS with a channel switching probability p.

### 5.2. Choice of the Channel Switch Probability p

The value of p can be set as a fixed value, for instance, p=1 (which means that the node will definitely switch its channel once the collision occurs) or p=0.5 (which means that the node has the same probability to stay on current channel or switch to another channel once the collision occurs). However, the fixed channel switching probability p cannot guarantee the node dwells on a channel of good condition considering the channel ranking, energy consumption and the influence of new nodes. In this research, we adopt a more efficient choice on p that it is not only related with the rank of channel condition (which leads the node to have higher probability to select the channel with better condition), but also corresponds with the time that a node stays on the current channel. Specifically, the selection of p should involve the following properties:

(i) p is related with the channel ranking so that each node will have higher opportunity to stay on the better channel and the network can rapidly converge to a collision-free channel allocation.
  - When the value of rd for next channel in the ACS is far from the value of the current channel’s rd, it means that the next channel is of poor condition and the node would prefer to stay on the current channel and it indicates p→0. Thus it can be modeled as p→0 when Δd→1 (where Δd is the difference between the rd of current channel and that of the next free channel in ACS).
  - On the other hand, when the rd of the next channel is close to that of the current channel rd, the node would prefer to switch the channel to avoid the collision, which means p→1 when Δd→0.
(ii) p is related with the residual energy so that each node can save energy.
  - When the residual energy is limited, the node would prefer to stay on the current channel to save energy, it indicates that p→0 when the consumed energy ratio $\Delta E=\frac{E-E_{r}}{E}\to 1$, where $E_{r}\;$ is the residual energy and E is the total energy.
  - With the decrease of energy, the energy should be the primary factor influencing p, thus the weight κ of residual energy will increase.
(iii) p is decreased with the dwelling time so that the new entrants will impose less impact on the old users.
  - When a node stays on a channel for a long time and we denote this state as t→∞, it will be less willing to switch channel and it indicates that p→0. In conclusion, p→0 when t→∞.
  - On the other hand, if a channel is new to this node, it will more likely to switch to another channel. That is when t=0, p only depends on Δd and $E_{r}\;.$

We select a simple model that satisfies these properties of p as follows,

(10) $$p={\left[f(\Delta d,\Delta E)\right]}^{g(t)}$$

where f(Δd,ΔE) reflects the influence of channel rank and residual energy, and g(t) reflects the influence of dwelling time. They are defined by

**Figure 4:**
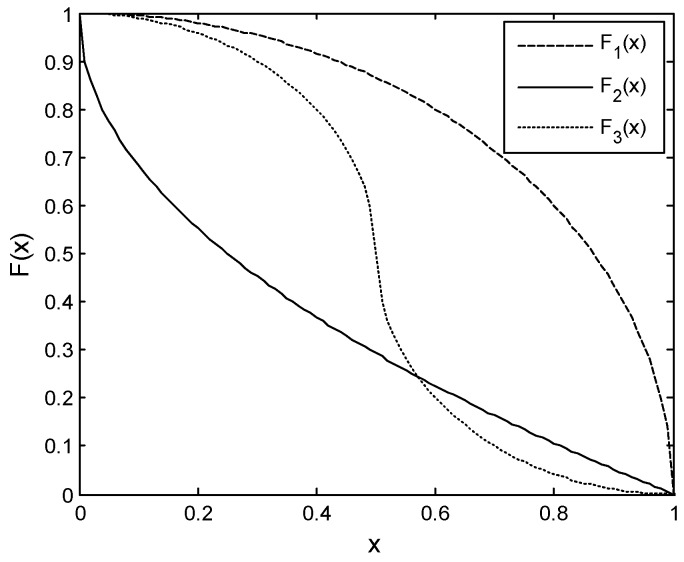
Three types of F(x).

(11) f(Δd,ΔE)=κF(ΔE)+(1−κ)F(Δd)

(12) g(t)=α·t+1

where κ represents the relative importance of residual energy to the channel quality (for simplification we set κ=ΔE in later numerical results); α is the inert factor and will be explained later; *t* is dwelling time (the time that a node continuously dwells on current channel, and once a node switches to a new channel it will reset *t* = 0)*.* F(x) is a decreasing function and in Figure 4, and we plot three representative curves of it, denoted by $F_{i}\left(x\right),\;i=1,\;2,\;3$ where

(13) $$F_{1}\left(x\right)=1-\sqrt{x^{2}}$$

(14) $$F_{2}\left(x\right)=\sqrt{\left(1-x^{2}\right)}$$

(15) $$F_{3}\left(x\right)=\{\begin{array}{c} 0.5+\sqrt{0.5^{2}-x^{2}},\;0\leq \;x\leq 0.5\\ 0.5-\sqrt{0.5^{2}-{\left(x-1\right)}^{2}},\;0.5<x\leq 1 \end{array}$$

The desirable condition is when Δd (or ΔE) is near to 0.5, the channel condition of the next channel (or this node’s residual energy) seriously deteriorates, and the switch probability should decline sharply. So we adopt $F_{3}\left(x\right)$ as F(x) in this research.

**Algorithm 1:** Simple distributed channel allocation1:  **Initialization:** 2:  Set up the available channel set (ACS) based on the channel ranking; 3:  Initialize t=0,  i=1;    // *i* is the channel ID in the ACS 4:  **Channel allocation:** 5:  *Thread A: Local information broadcast* 6:   **While** (Hello Timeout) **do** 7:    Broadcast HELLO on Channel (*i*), which encodes its own ID; 8:    t=t+1; 9:   **end while** 10: *Tread B: Channel switching* 11:   **While** (Received a HELLO packet on current channel) **do** 12:    Calculate p according to (10) 13:    if rand(1)≤p 14:     Set i=(i+1)mod (M);  //Switch channel with the probability of p 15:     t=0;         //Reset t after switching the channel 16:    end if17:  **end while**

**Figure 5:**
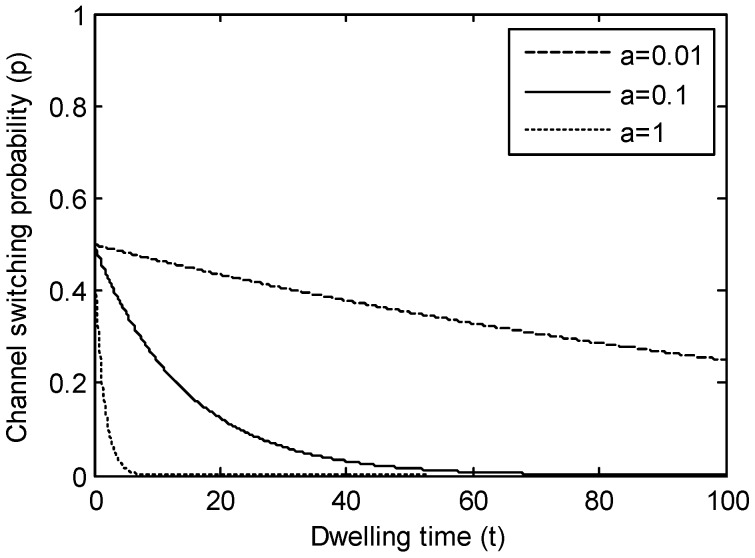
Channel switch probability under different α.

The selection of p will result in elder nodes dwelling on one channel becoming more willing to stay at this channel while new entrants becoming more willing to jump to a new channel. And this property contributes to fast convergence and stable network that new entrants will affect little the existing nodes. To consider the effect of dwelling time, in Figure 5, we plot the curves of g(t) in (12) with different values of α under f(Δd,ΔE)=0.5, which shows that α=0.1 is a proper choice under the scenarios considered in this research.

The distributed channel allocation algorithm is shown in Algorithm 1 which is composed of two parallel threads. Note that Thread A is a periodical thread to broadcast HELLO packet and accumulate the channel dwell time. Thread B is an interrupt thread triggered by receiving a HELLO packet. And in this thread, each node will decide which channel (e.g., the current channel or another channel) to dwell based on the calculation of p.

## 6. Analysis and Optimization on Energy Consumption

**Figure 6:**
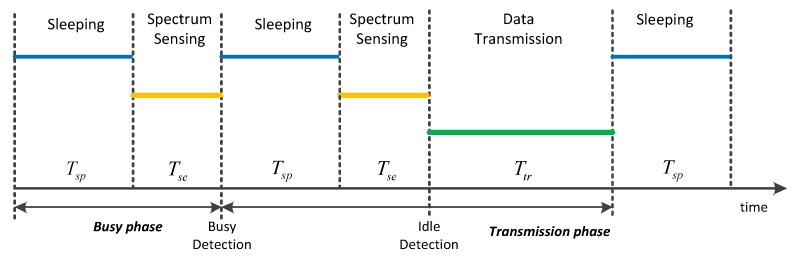
The part of slotframe structure.

After the node has chosen the channel, it needs to determine its behavior based on the residual energy. When the current battery of the node is insufficient, it needs to sleep periodically to prolong its lifetime. Thus, the node will perform three operations: sleeping, sensing and data transmission as shown in shown in Figure 6 [16]. The lengths of these three operations are respectively denoted by $T_{sp}$, $T_{se}$ and $T_{tr}$, and the power consumed during these three operations are respectively denoted by $P_{sp}$, $P_{se}\;$ and $P_{tr}$. The energy consumed in the data transmission period is certain once the channel has been chosen, given the packet size. Therefore, the main motivation of this part is to adjust the lengths of sleeping and sensing operations to ensure the normalized throughput while minimize the energy consumption.

Two probabilities reflect the node performance during the sensing period: the probability of detection ($P_{d}$), which is the probability that one node can correctly detect the interference signal, and the probability of false alarm ($P_{f}$), which is the probability that the node falsely declares the presence of interference signal. These two probabilities are calculated as follows [16].

(16) $$P_{d}\left(T_{se},\varepsilon \right)=Q\left(\left(\frac{\varepsilon }{N_{0}}-\gamma -1\right)\sqrt{\frac{T_{se}\cdot f_{s}}{2\gamma +1}}\right)$$

(17) $$P_{f}\left(T_{se},\varepsilon \right)=Q\left(\left(\frac{\varepsilon }{N_{0}}-1\right)\sqrt{T_{se}\cdot f_{s}}\right)$$

where $Q\left(x\right)=\frac{1}{\sqrt{2\pi }}\int_{x}^{\infty }e^{-t^{2}/2}dt$. The detection probability is decided by the detection threshold ε and the sampling frequency $f_{s}$. γ is the signal to noise ratio (SNR). The noise is a Gaussian random process with mean zero and variance $N_{0}$. We can see that these two probabilities are all influenced by the spectrum sensing time $T_{se}$.

As illustrated in Figure 6, the “busy phase” consists of sleeping period and sensing period. Assuming $p_{i}$ and $p_{b}$ are the probabilities of the idle and the busy channel respectively, we can calculate the probability of the busy phase appearance as

(18) $$P_{S}\left(T_{se},\varepsilon \right)\;=\;p_{i}P_{f}\left(T_{se},\varepsilon \right)\;+\;p_{b}P_{d}\left(T_{se},\varepsilon \right)$$

Similarly we can calculate the probability of the transmission phase appearance as follows,

(19) $$P_{T}(T_{se},\varepsilon )=\;P_{T1}(T_{se},\varepsilon )+P_{T2}(T_{se},\varepsilon )=p_{i}(1-P_{f}(T_{se},\varepsilon ))\;+\;p_{b}(1-P_{d}(T_{se},\varepsilon ))$$

The first item $P_{T1}$ represents the case that the channel is idle and correctly detected, and second item $P_{T2}$ represents the case that the channel is busy but wrongly judged as idle. 

Considering the practical scenario, one node may go through successive *k* times of the busy phase before data transmission, and the probability of this condition is

(20) $$P_{T}\left(k,T_{se},\varepsilon \right)\;=\;P_{T1}(k,T_{se},\varepsilon )+P_{T2}(k,T_{se},\varepsilon )=P_{S}{\left(T_{se},\varepsilon \right)}^{k}\cdot P_{T1}\left(T_{se},\varepsilon \right)+P_{S}{\left(T_{se},\varepsilon \right)}^{k}\cdot P_{T2}\left(T_{se},\varepsilon \right)$$

The node will be influenced by the interference signal with the power $P_{n}$, and $N_{i}$ is the noise power. When the channel is idle, the transmission rate is $R_{1}=B\cdot \log_{2}\left(1+\frac{P_{tr}}{N_{i}}\right)$, and when there is interference, the transmission rate is $R_{2}=B\cdot \log_{2}\left(1+\frac{P_{tr}}{P_{n}+N_{i}}\right)$.

Given the packet length *L*, the normalized throughput can be expressed as:

(21) $$S\left(T_{sp},T_{se},\varepsilon \right)=\sum_{k=0}^{\infty }P_{T1}\left(k,T_{se},\varepsilon \right)S_{1}\left(k,T_{sp},T_{se}\right)\text{​}+\text{​}P_{T2}\left(k,T_{se},\varepsilon \right)S_{2}\left(k,T_{sp},T_{se}\right)$$

where $S_{1}$ and $S_{2}$ are the average throughputs with no interference and with interference respectively,

(22) $$S_{1}\left(k,T_{sp},T_{se}\right)=\frac{L}{\left(k+1\right)\left(T_{sp}+T_{se}\right)+L/R_{1}}$$

(23) $$S_{2}\left(k,T_{sp},T_{se}\right)=\frac{L}{\left(k+1\right)\left(T_{sp}+T_{se}\right)+L/R_{2}}$$

As the node energy supply is limited, it is particularly important for node to sleep to reduce the node energy consumption and to extend the network lifetime. We assume that the total remaining energy is ΔE, and the expected survival time is $T_{0}$, thus the maximum consuming power is $\bar{P_{d}^{max}}=\frac{\Delta E}{T_{0}}$. And the average power consumption can be expressed as:

(24) $$\bar{P}\left(T_{sp},T_{se},\varepsilon \right)=\frac{P_{T1}\left(k,T_{se},\varepsilon \right)\cdot \left[(k+1)\cdot (P_{sp}T_{sp}+P_{se}T_{se})+P_{tr}\cdot L/R_{1}\right]}{(k+1)\cdot (T_{sp}+T_{se})+L/R_{1}}+\frac{P_{T2}\left(k,T_{se},\varepsilon \right)\cdot \left[(k+1)\cdot (P_{sp}T_{sp}+P_{se}T_{se})+P_{tr}\cdot L/R_{2}\right]}{(k+1)\cdot (T_{sp}+T_{se})+L/R_{2}}$$

The objective is to enhance the lifetime of node through sleeping while guarantying the normalized throughput. Therefore, we can formulate the optimization problem as

**Problem** **1.** $$\underset{T_{sp},T_{se},\varepsilon }{\mathrm{maximize}}\;S\left(T_{sp},T_{se},\varepsilon \right)$$

$$subject\;to\;P_{d}\left(T_{se},\varepsilon \right)\geq \bar{P_{d}}$$

(25) $$\bar{P}\left(T_{sp},T_{se},\varepsilon \right)\leq \bar{P_{d}{}^{\max}}$$

*where* $\bar{P_{d}}$ *is the detection probability threshold. The remaining problem is to find the optimal combination of sleeping time and sensing time.*

**Theorem** **1.** *When the combination of* $T_{se}\;and\;\varepsilon$ *can satisfy* $\;P_{d}\left(T_{se}^{*},\;\varepsilon^{*}\right)=\bar{P_{d}}$*, it is the optimal solution for Problem 1.*

**Proof.**  We provide a proof by contradiction to Theorem 1.

We assume that the combination of $T_{sp}^{0},\;T_{se}^{0},\;\varepsilon^{0}$ can maximize $S\left(T_{sp},\;T_{se},\;\varepsilon \right)$ and it meet the condition of $P_{d}\left(T_{se}^{0},\;\varepsilon^{0}\right)\geq \bar{P_{d}}$. The probability of detection and the probability of false alarm decrease with the detection threshold ε increase. There exists a larger $\varepsilon^{1}$, i.e., $\varepsilon^{1}\geq \varepsilon^{0}$, which can satisfy $P_{d}\left(T_{se}^{0},\;\varepsilon^{1}\right)=\bar{P_{d}}$ and $P_{f}\left(T_{se}^{0},\;\varepsilon^{0}\right)>P_{f}\left(T_{se}^{0},\;\varepsilon^{1}\right)$. The decrease of probability of detection and the probability of false alarm will lead to higher data transmission rate, which is $S\left(T_{sp}^{0},\;T_{se}^{0},\varepsilon^{1}\right)>S\left(T_{sp}^{0},\;T_{se}^{0},\varepsilon^{0}\right)$ and it contradicts the assumption. Therefore, when $P_{d}\left(T_{se}^{*},\;\varepsilon^{*}\right)=\bar{P_{d}}$, the combination of $T_{se}\;\mathrm{and}\;\varepsilon$ can optimized the average throughput. ☐

Then we further explore the property of $S\left(T_{sp},\;T_{se},\;\varepsilon \right)$ in terms of $T_{sp}$, $T_{se}$ and ε. First, we consider the impact of $T_{sp}$. For a given $T_{se}^{0}$, we can solve (16) to compute $\varepsilon^{0}$. It is easy to demonstrate that the normalized throughput decreases with $T_{sp}$ based on (22) and (23). 

Next, we derive the impact of $T_{se}$. As the $\bar{P_{d}}$ is chosen from [0,1], we choose $\bar{P_{d}}=0.9$ as an example. Also, we suppose the probability of $p_{b}$ is small ($p_{b}\leq 0.2$). Moreover, since $R_{1}>R_{2}$, the first term in Equation (21) dominates the normalized throughput. Therefore, for a given $T_{sp}^{0}$, Problem 1 can be firstly converted into

**Problem** **2.** $$\underset{T_{sp},T_{se},\varepsilon }{\mathrm{maximize}}\tilde{S}\left(T_{se}\right)=P_{T1}\left(T_{se},\varepsilon \right)S_{1}\left(T_{se}\right)$$

(26) $$subject\;to\;P_{d}\left(T_{se},\varepsilon \right)\geq \bar{P_{d}}$$

*When the detection probability threshold* $\bar{P_{d}}$ *is determined, we can transform* $P_{f}$ *as follows based on (17) and (18),*

(27) $$P_{f}=Q\left(\sqrt{2\gamma +1}Q^{-1}(\bar{P_{d}})+\sqrt{T_{se}\cdot f_{s}}\gamma \right)$$

And we can derive $\tilde{S}(T_{se})$ as

(28) $$\tilde{S}(T_{se})=P_{T1}\left(T_{se},\varepsilon \right)\cdot S_{1}\left(T_{se}\right)=p_{i}(1-P_{f}(T_{se},\varepsilon ))\cdot \frac{L}{T_{se}+T_{sp}+L/R_{1}}=p_{i}\cdot (1-Q\left(\alpha +\sqrt{T_{se}\cdot f_{s}}\gamma \right))\cdot \frac{L}{T_{se}+\beta }$$

where $\beta =T_{sp}+L/R_{1}$, $\alpha =\sqrt{2\gamma +1}Q^{-1}(\bar{P_{d}})$. From (28), we can find that the normalized throughput is a function of $\;T_{se}$.

**Theorem** **2.** *For any detection probability threshold* $\bar{P_{d}}$ *, there exists an optimal sensing time to get the maximum normalized throughput.*

**Proof.**  Detailed proof is given in Appendix A. ☐

From Theorem 2, we can conclude that there exists a unique solution to get the maximum normalized throughput. The next step is to identify whether the optimal solutions can satisfy the power consumption restraint in Equation (25). Exhaustive search is needed in order to find the optimal solution. We vary the value of $T_{sp}$, and compute the corresponding normalized throughput (see Equation (20)). We take one-dimensional exhaustive search on $T_{sp}$ for example as described in Algorithm 2, which can be adopted to obtain the optimal solution.

**Algorithm 2:** Optimization for $T_{sp}$1:  **Initialization:** 2:  Set up the range of $T_{sp}\in \left[t_{sp\_min},\;t_{sp\_max}\right]$; 3:  Initialize the optimal solution $t_{sp}^{*}=0$; 4:  **Optimization calculation:** 5:   **While** ($t_{sp\_max}-t_{sp\_min}>1$) **do** 6:    $t_{sp}^{now}$ = $\left\lfloor \frac{t_{sp\_max}+t_{sp\_min}}{2}\right\rfloor$ 7:    compute the average power $\bar{P}\left(t_{sp}^{now}\right)$ based on (24); 8:    **if**
$\bar{P}\left(t_{sp}^{now}\right)\leq \bar{P_{d}^{max}}$
**then** 9:      compute the average throughputs $S\left(t_{sp}^{now}\right)$ based on (21); 10:      $t_{sp}^{next}=t_{sp}^{now}+1$; 11:      compute the average power $\bar{P}\left(t_{sp}^{next}\right)$ based on (24); 12:     **if**
$\bar{P}\left(t_{sp}^{next}\right)\leq \bar{P_{d}^{max}}\;$
**then** 13:       compute the average throughputs $S\left(t_{sp}^{next}\right)$ based on (21); 14:       **if**
$S\left(t_{sp}^{now}\right)\leq S\left(t_{sp}^{next}\right)$
**then** 15:       
 $t_{sp\_min}=t_{sp}^{now}$, $t_{sp}^{*}=t_{sp}^{next}$; 16:       **else if**
$S\left(t_{sp}^{now}\right)>S\left(t_{sp}^{next}\right)$
**then** 17:        $\;t_{sp\_max}=t_{sp}^{now}$, $t_{sp}^{*}=t_{sp}^{now}$; 18:       **end if** 19:     **end if** 20:    **end if** 21:   **end while**

## 7. Simulation Result and Discussion

### 7.1. Simulation Setup

**Figure 7:**
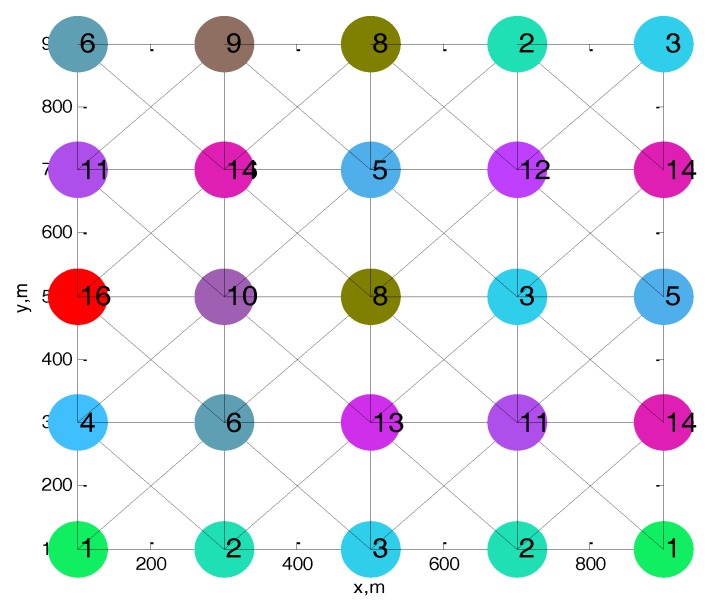
Simulation network topology.

The simulation tool in this paper is MATLAB 2015b. We take the grid topology for example in the following simulations as shown in Figure 7 based on the typical application scenarios in IoT, the grid topology pattern include: (i) crops monitoring in farmland; (ii) facilities’ states monitoring for safety; (iii) monitoring of water surface; and (iv) fire or animal monitoring in grassland [31]. Moreover, our proposed solution can also apply in random topology. We use grid-topology network rather than random network, because under the grid-topology network, we can intentionally control the interference among nodes, especially when new nodes entering the network, and therefore we can better evaluate the performance of our proposed scheme under different scenarios. We assume that the number of available channels is 16. The attributes of each channel is set as shown in the Table 2, the transmission power is allocated based on [11], thus the channel condition of each node is different. The interference range is 600 m. The dotted-lines connecting the nodes represent two nodes within the interference range, and the number on the node represents the dwelling channel of node. The node in the center will be interfered with 20 nodes at most with the increase in the density. Figure 7 shows that the heuristic algorithm can achieve collision-free channel allocation.

The ranking of channels is based on variable parameters, considering the time-varying channels. We assume the maximum speed of node is 2 m/s. Simulation parameters of channel attributes are listed in Table 2 [11].

We consider the slotframe is composed of 100 timeslots. ED period occupies 5 timeslots, and channel allocation occupies 10 timeslots. The rest of 85 timeslots are used for sensing, sleeping and transmission. Simulation parameters are shown in Table 3. The noise power $N_{i}$ is −50 dB and the sleeping power $P_{sp}$ and spectrum sensing power $P_{se}$ is 0.1 mW and 2 μW respectively [16].

### 7.2. Simulation Results

(i) Convergence property

**Figure 8:**
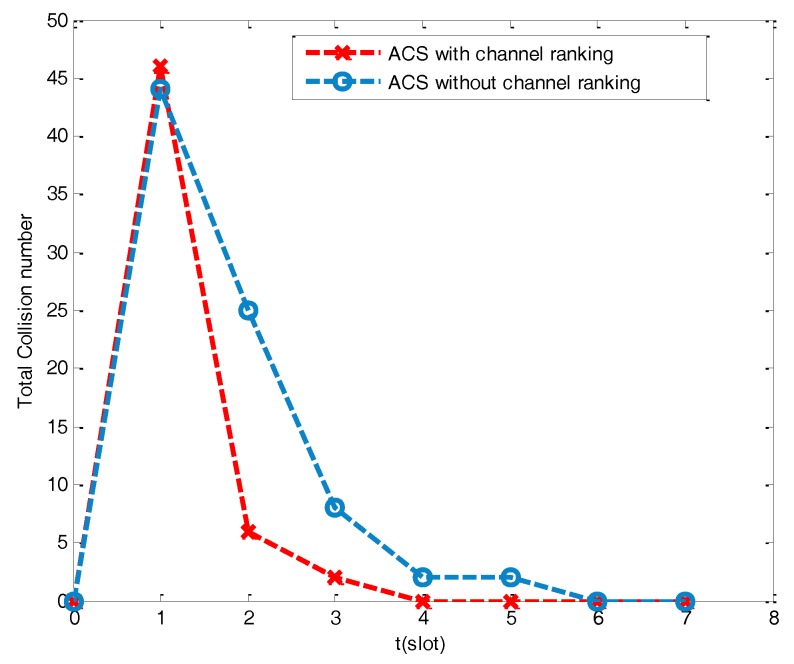
Convergence time under different available channel set (ACS).

The convergence property can be observed when the network obtains a collision-free channel allocation. In research [32], each node’s ACS is randomly oriented without the channel condition ranking. As each channel is identical, the node has the same probability to stay on the current channel or randomly switch to another channel once collision occurs. In Figure 8, the network topology is made up of 50 nodes, and the channel allocation is combined with channel ranking to make a significant difference in the convergence time when contrasted with ACS irrespective of channel ranking. ACS combined with channel ranking only needs 4 slots to finish the channel allocation of 46 collision nodes, while ACS without channel ranking needs 6 slots to finish the channel allocation of 44 collision nodes. The former one has fast convergence property as the *rd* of the channel is to act as a guide to make a distinction so that the nodes are more likely to be allocated with different channels.

(ii) Satisfaction of the allocated channel 

**Figure 9:**
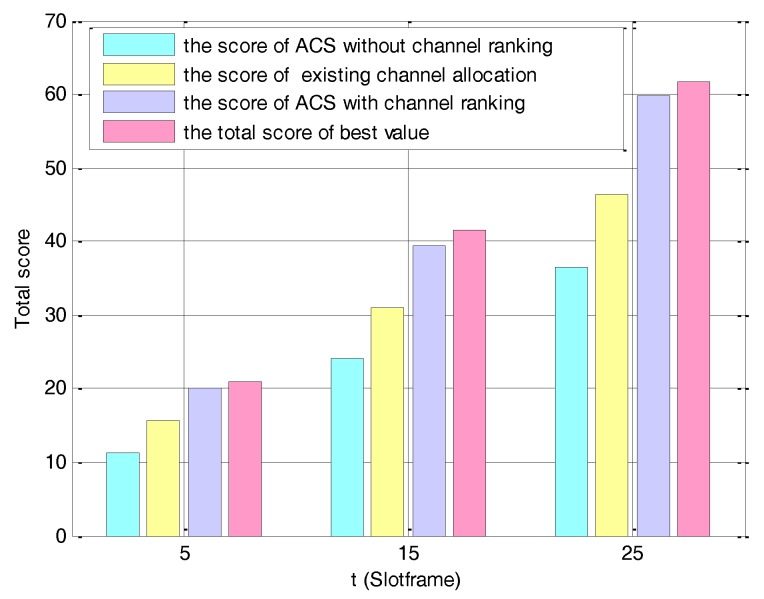
Score under different channel allocation algorithms.

In order to estimate the satisfaction degree of each node on its allocated channel, we define a satisfying score and compare the score obtained by our proposed algorithm to those obtained by existing works. In Figure 9, the score is the sum of the rd of the final allocation channel of each node. The total score of the best value is the sum of the highest score of each node’ channel according to the channel ranking, and it can be computed as,

(29) $$total\;score=\sum_{j=1}^{N}\arg\;\max rd_{ij}\;\forall i\in [1,m]$$

where N and m represent the numbers of nodes and channels respectively.

We calculate the score under different slotframes with another 25 nodes join the network in slotframe 15th and 25th as shown in Figure 9. The score of the ACS with the channel ranking is apparently higher than existing works [32,33]. This is because that existing works simply classify the channels into two categories, i.e., good channels and poor channels, and then randomly allocate the good channels to the nodes. Thus, the nodes can only use channel with relative good quality instead of the most ideal channel. The score of the ACS with the channel ranking is sufficiently close to the total score of the best value, which means that this algorithm allows allocating nodes to the most ideal channel for the greatest level.

(iii) Effect of time slot optimization

**Figure 10:**
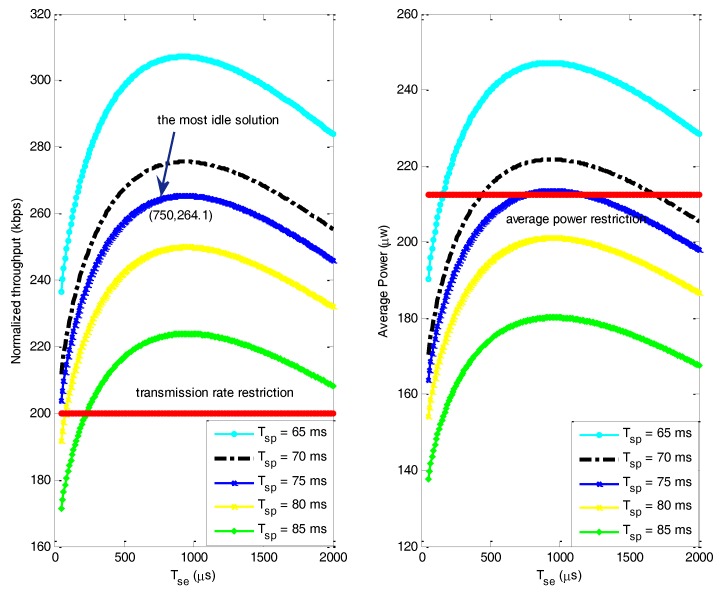
The normalized throughput under different sleeping durations and spectrum sensing durations.

Figure 10 shows the normalized throughput under different spectrum sensing time and sleeping time and presents a combination of spectrum time and sleeping time which maximizes the normalized throughput while satisfying the power constraint separately. The left and right red line in Figure 10 represents the constraint of the normalized throughput and the power separately. The normalized throughput should be no less than the constraint and the consumption power should be no more than the constraint. We can observe that the normalized throughput increases before spectrum sensing time reaches the peak and then decreases. Therefore, the most ideal solution is when $T_{se}=750\;\mu s\;$ and $T_{sp}=75\;\mathrm{ms}$, the maximum average transmission rate can reach 264.1 kbps. The simulation result also validates the feasibility of Algorithm 2. 

**Figure 11:**
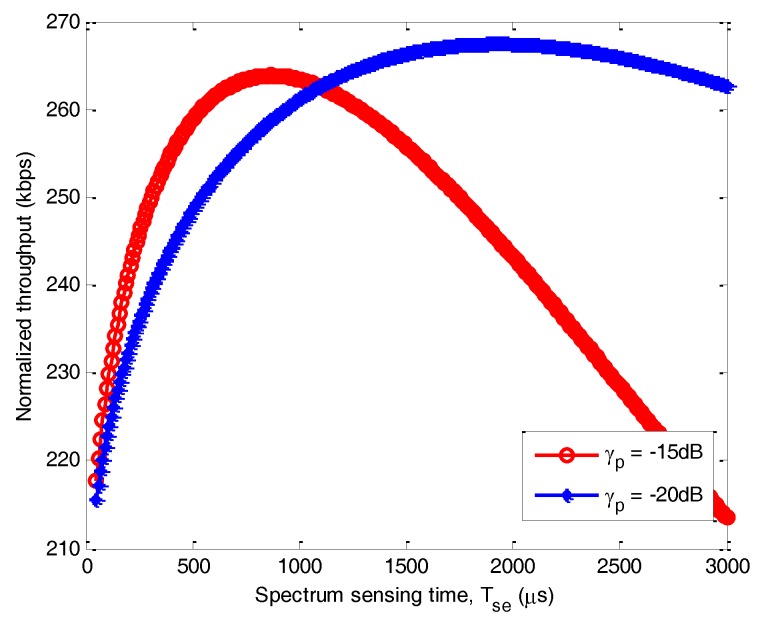
Variation of the normalized throughput under different γ.

Figure 11 shows the variation of the normalized throughput under different value of γ. When γ increases from −20 dB to −15 dB, the normalized throughput decreases. The increase of γ improves the interference to transmission, resulting in the increase of transmission power and thus the sleeping time need to prolong to satisfy the power constraint. In addition, it is easier for node to detect the channel state with increasing γ, so the spectrum sensing duration has been decreased. It also corresponds with the idea of channel allocation that the node with less residual energy needs to choose the channel with high quality to save energy.

(iv) Energy saving

**Figure 12:**
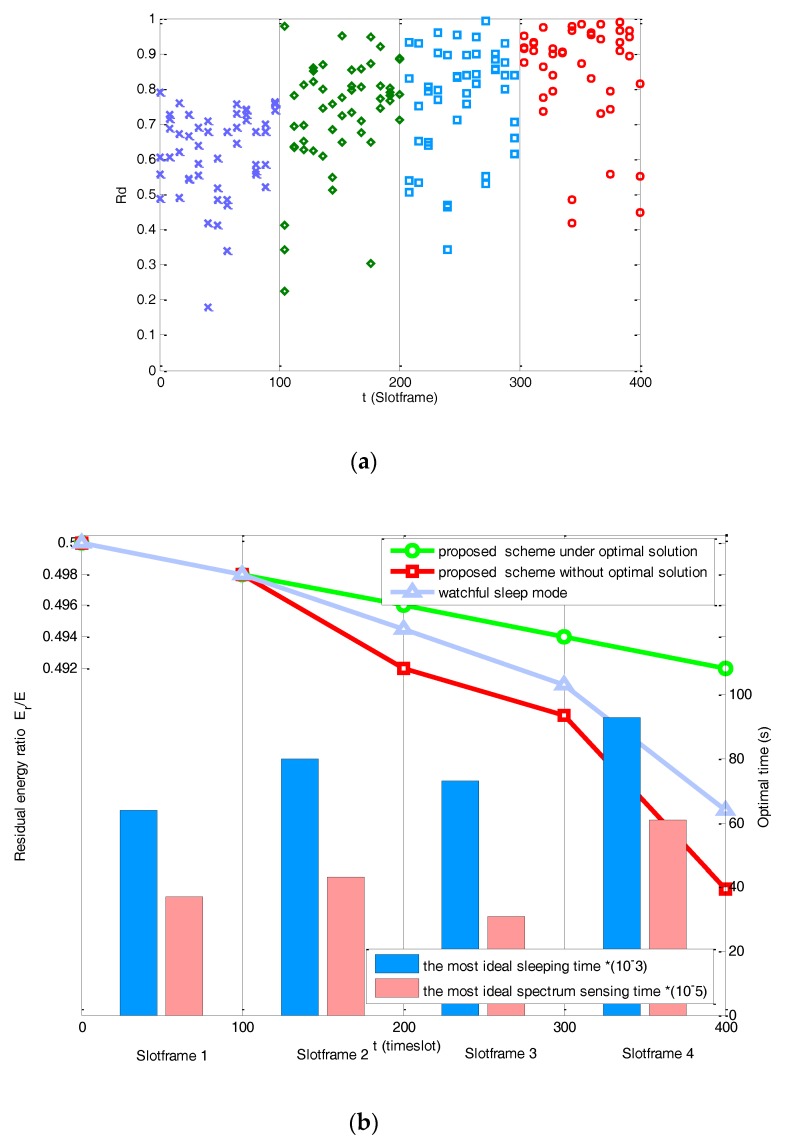
Joint energy saving in frequency and time domain*.* (**a**) Energy saving in frequency domain; (**b**) energy saving in time domain.

As the energy saving is a joint optimization in both frequency domain and time domain, i.e., via channel selection and time slot allocation. We first observe the energy saving in frequency domain as shown in Figure 12a. We present the *rd* of the nodes’ dwelling channel under different slotframes. The *rd* represents the selected channel under the variation of p. As the residual energy is decreasing with time goes by, we can observe that the nodes with lower residual energy are more likely to be allocated on the channel with good condition under the influence of p. Therefore, the extension of the battery lifetime can better enable the connectivity of IoT.

On the other hand, Figure 12b reflects the energy saving in time domain when compared with the existing work [34]. Researchers in [34,35] propose a Watchful Sleep mode, which periodically switches off some of its components to save energy. As the algorithm in [34,35] can enter a Low Power Watch state, where it may have its receiver and/or transmitter OFF, it is more energy efficient comparing to our algorithm without optimal time slot. The reason is that fixed sleeping time and spectrum sensing time without optimizing cannot deal with the variable channel condition. However, after adopting the optimization algorithm, our proposed scheme can save more energy than existing works.

The main reason is that our solution can adapt to different channel condition. When the channel condition is worse during the slotframes 2–4 in Figure 12b, and results in the more transmission power, then the energy constraint will be set more stringent to prolong the lifetime of node. The optimal sleeping time and spectrum sensing time can guarantee the energy decreases steadily and it is dynamic during different slotframes as shown in Figure 12b. We can observe that the sleeping period has been extended to deal with the strict power constraint.

**Figure 13:**
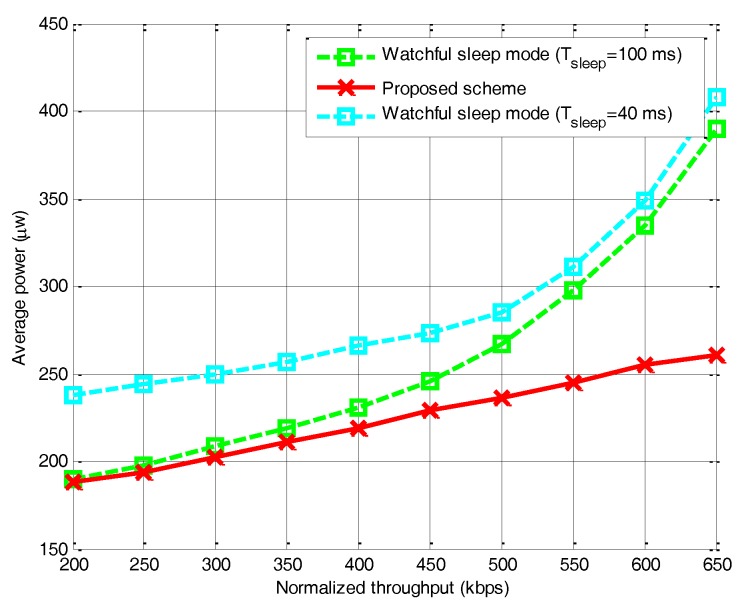
Comparison of the average power*.*

Then we evaluate the performance of average power consumption under different normalized throughput, and compare it with the Watchful Sleep mode in Figure 13. We first performed simulations for $T_{\mathrm{sleep}}=100\;\mathrm{ms}$ and 40 ms, in accordance with $T_{\mathrm{aware}}=5\;\mathrm{ms}$ and $T_{\mathrm{watch}}=1\;s$ based on [35]. With lower traffic arrival rate, the Watchful Sleep mode with longer sleep period indeed decreases the average power consumption. However, with the increase of traffic arrival rate, the average power increases sharply. On the other hand, in our proposed scheme, the sleeping time and spectrum sensing time are adaptive and always keep the optimal values, and therefore consume the least energy. We can conclude that the optimal solution can not only control the power consumption but also maximum the normalized throughput.

## 8. Conclusions

In this paper, we consider the problem of alleviating the interference and energy saving of the node. To address this problem, we propose a joint channel allocation and time slot optimal algorithm for high-density IoT. The algorithm ranks channels, and hence, selects the optimal channel for the node, and a learning process is proposed to ensure the node can adapt its channel switching strategy based on its residual energy and dwelling time. Furthermore, we propose a dynamic time-slot structure to jointly optimize the sleeping time and the spectrum sensing time. Simulation results illustrate that joint optimization in both frequency domain and time domain have following advantages: (i) the network can rapidly converge to a collision-free transmission, meanwhile ensure that each node can be allocated to a proper channel; and (ii) the optimal solution maximize the node’s throughput while satisfying the energy consumption constraint. We believe this flexible framework is efficient and adaptive to the high-density multi-channel IoT networks. In future work, we will investigate the effectiveness of the solution in real-world testbeds.

## Figures and Tables

**Table 1:** Linguistic Scales of Importance.

Relative Importance of Attribute *i* to Attribute *j*	$b_{ij}$
Equal importance	1
Moderate importance of one factor over another	3
Strong or essential importance	5
Very strong importance	7
Extreme importance	9
middle state between adjacent two stages	2, 4, 6, 8

**Table 2:** The distributions of each channel attribute.

Channel Attribute	Mathematical Distribution
SINR	Uniform distribution of 5–30 db
Bandwidth	Random distribution in 200 kHz–1 MHz
Coherent bandwidth	Delay spread obey uniform distribution of 5–20 kHz
Coherent time	Random distribution in 18–21 ms
Energy consumption	Same as [11] (lower transmission power in flat channel and more transmission power in fading channel)

**Table 3:** Simulation parameters.

Description	Value
Sampling frequency $f_{s}$	1 MHz
Available bandwidth B	1 MHz
Idle probability of the channel $p_{i}$	0.8
Busy probability of the channel $p_{b}$	0.2
Spectrum sensing power $P_{se}$	2 μW
Sleeping consuming power $P_{sp}$	0.1 mW
Transmission power $P_{tr}$	20 mW
Detection probability threshold $P_{d}$	0.9
Length of data packet L	105 bits

## Appendix A. Proof of Theorem 2

From (28), we can derive the differentiation of $\tilde{S}(T_{se})$,

(A1) $${\tilde{S}}^{\prime }(T_{se})=p_{i}\cdot [-\frac{L}{{\left(T_{se}+\beta \right)}^{2}}(1-Q\left(\alpha +\sqrt{T_{se}\cdot f_{s}}\gamma \right))+\frac{L}{T_{se}+\beta }\cdot \frac{\gamma \sqrt{f_{s}}}{2\sqrt{2\pi \cdot T_{se}}}\cdot \exp(-\frac{{\left(\alpha +\sqrt{T_{se}\cdot f_{s}}\gamma \right)}^{2}}{2})]$$

Obviously,

(A2) $$\underset{T_{se}\to 0}{\lim}{\tilde{S}}^{\prime }(T_{se})=+\infty$$

(A3) $$\underset{T_{se}\to \infty }{\lim}{\tilde{S}}^{\prime }(T_{se})<L\cdot T_{se}{}^{2}\cdot (-1+\frac{\gamma \sqrt{f_{s}}}{2\sqrt{2\pi }}\cdot T_{se}{}^{1/2}\exp(-\frac{\gamma^{2}f\cdot T_{se}}{2}))<0$$

Equations (A2) and (A3) mean that $\tilde{S}\left(T_{se}\right)$ increases when $T_{se}$ is small, and then decreases when $T_{se}$ get larger. We further show that $\tilde{S}\left(T_{se}\right)$ is a concave function in $T_{se}$ to make an unique maximum point.

In Appendix B, we further show that the concavity of $\tilde{S}\left(T_{se}\right)$.

## Appendix B. Concavity of $\tilde{S}\left(T_{se}\right)$

**Theorem** **A1.** *An unique optimal sensing time of* $\tilde{S}\left(T_{se}\right)$ *exists when* $P_{f}\left(T_{se}\right)<0.5$*.*

**Proof.** First, derive the differentiation of $P_{f}$ in (27),

(A4) $$P_{f}{}^{\prime }(T_{se})=-\frac{\gamma \sqrt{f_{s}}}{2\sqrt{2\pi \cdot T_{se}}}\cdot \exp(-\frac{{\left(\alpha +\sqrt{T_{se}\cdot f_{s}}\gamma \right)}^{2}}{2})$$

One can see that $T_{se}>0$, $P_{f}^{\prime }\left(T_{se}\right)<0$, which means that $P_{f}\left(T_{se}\right)$ is decreasing with $T_{se}$. Moreover, when $P_{f}\left(T_{se}\right)<0.5$, $\alpha +\sqrt{T_{se}\cdot f_{s}}\gamma \geq 0$. It demonstrates that $P_{f}\left(T_{se}\right)$ is convex when $P_{f}\left(T_{se}\right)<0.5$. Therefore, based on Theorem A1, we can conclude that ${\tilde{S}}^{\prime }\left(T_{se}\right)$ is decreasing with $T_{se}$, and thus $\tilde{S}\left(T_{se}\right)$ is a concave function of $T_{se}$. ☐
